# The Initiation of Th2 Immunity Towards Food Allergens

**DOI:** 10.3390/ijms19051447

**Published:** 2018-05-12

**Authors:** Yosef Ellenbogen, Rodrigo Jiménez-Saiz, Paul Spill, Derek K. Chu, Susan Waserman, Manel Jordana

**Affiliations:** 1 McMaster Immunology Research Centre (MIRC), Department of Pathology & Molecular Medicine, McMaster University, MDCL 4013, 1280 Main St W, Hamilton, ON L8N 3Z5, Canada; yosef.ellenbogen@medportal.ca (Y.E.); jimenez@mcmaster.ca (R.J.-S.); spillp@mcmaster.ca (P.S.); 2 Department of Medicine, McMaster University, Hamilton, ON L8N 3Z5, Canada; chudk@mcmaster.ca (D.K.C.); waserman@mcmaster.ca (S.W.)

**Keywords:** Th2 immunity, food allergy, allergic sensitization, allergens, alarmins, initiation of allergy, IgE, allergic disease

## Abstract

In contrast with Th1 immune responses against pathogenic viruses and bacteria, the incipient events that generate Th2 responses remain less understood. One difficulty in the identification of universal operating principles stems from the diversity of entities against which cellular and molecular Th2 responses are produced. Such responses are launched against harmful macroscopic parasites and noxious substances, such as venoms, but also against largely innocuous allergens. This suggests that the established understanding about sense and recognition applied to Th1 responses may not be translatable to Th2 responses. This review will discuss processes and signals known to occur in Th2 responses, particularly in the context of food allergy. We propose that perturbations of homeostasis at barrier sites induced by external or internal subverters, which can activate or lower the threshold activation of the immune system, are the major requirement for allergic sensitization. Innate signals produced in the tissue under these conditions equip dendritic cells with a program that forms an adaptive Th2 response.

## 1. Introduction

Th2 responses are generated against structurally diverse entities, including macroscopic parasites, noxious substances (e.g., poisons and venoms), and largely innocuous antigens, such as food allergens [1,2]. Therefore, it is difficult to delineate universal operating principles governing their development. The diversity of entities against which Th2 responses are generated also suggests that the established understanding regarding the sense and recognition of Th1 responses to viruses and bacteria is likely not translatable to Th2 responses. Furthermore, as noted by Netea et al., the initiation of Th2 immunity requires the engagement of the entire tissue including not only immune cells but also tissue structural cells, such as epithelial cells [3]. Additionally, deciphering the signals that emerge from the tissue microenvironment may provide insights about the initiation of Th2 responses. This review will discuss processes and signals that initiate Th2 immunity in the context of food allergy. First, we introduce what is known about the acquisition of a Th2 identity. Second, we discuss the extent to which food allergens are innocuous, concluding that their inherent immunogenicity likely plays only a minor role. Thus, we propose that concomitant events that subvert the steady state of the microenvironment produce signals that prime the immune surveillance system to react to allergens present at the site. Third, we consider signals known to be produced at barrier sites (skin and mucosa) that equip dendritic cells (DCs) with the ability to facilitate the generation of Th2 immunity. Fourth, we introduce the concept that elicitation of Th2 responses to largely harmless allergens may also occur as a result of specific internal, i.e., genetic, alterations that decrease the threshold of activation of the immune system. Ultimately, we argue that allergen-independent, concomitant perturbations that are either internal and/or externally driven subvert tissue homeostasis and play the predominant role in the facilitation of Th2 responses to food allergens.

## 2. On the Acquisition of a Th2 Identity

In the late 1980s, Mosmann et al. transformed the understanding of CD4 T cells by both classifying them based on their cytokine profile upon stimulation and demonstrating their unique functionalities [4]. Essentially, Th1 cells generated IFN-γ, while Th2 cells produced IL-4 (once referred to as B-cell stimulating factor-1, BSF-1) and IL-5. Subsequently, it became clear that Th1 cells predominated in responses against viruses and intracellular bacteria, while Th2 cells were required for those against extracellular pathogens. It was also proposed that a Th1/Th2 imbalance towards Th2 could be the cause of allergic diseases [5,6]. These findings prompted studies to elucidate mechanisms through which naïve CD4 T cells could acquire a Th2 phenotype.

In vitro experiments with naïve CD4 T cells subjected to polyclonal activation showed that IL-2 and IL-4 were critical for Th2 polarization [7,8]. However, two factors, namely, the role of IL-2 in facilitating T helper differentiation of various subsets (e.g., Th1, Th2, inducible Tregs.) [9] in addition to the identification of IL-4 as the major product of, and specific for Th2 cells, highlighted that Th2 differentiation might involve an IL-4 positive feedback loop [10]. William Paul’s group investigated this concept in a co-culture system of CD4 T cells, with a transgenic TCR specific for pigeon cytochrome C or chicken egg white ovalbumin (OVA) and myeloid cells. They found that low concentrations of cognate peptide induced an “early” IL-4 production that was IL-4-independent and required IL-2 mediated STAT5 activation and GATA3 induction. IL-4 signalling further up-regulated GATA3 expression via STAT6 phosphorylation and completed the Th2 differentiation process [11]. Therefore, the incipient events of Th2 polarization of a naïve CD4 T cell do not require IL-4 signalling; rather, they depend on GATA3 expression.

In vivo studies demonstrated that robust Th2 polarization could occur in an IL-4 independent manner under some circumstances [12] but still required GATA3 [13,14], which became the master regulatory transcription factor for Th2 differentiation. However, the role of IL-4 is crucial for the comprehensive manifestation of Th2 responses, including the generation of humoral immunity. As such, mice deficient in IL-4 [15], IL-4Rα [16], or STAT6 [17,18,19] do not produce significant IgE and exhibit a dramatic reduction in serum levels of IgG1 following nematode infection or anti-IgD immunization in comparison with their wild-type counterparts. These findings are of particular interest for IgE-mediated diseases such as food allergy [20]. In this regard, in a model of peanut allergy, we reported that B-cell- or CD40L-deficient mice could be sensitized, as indicated by production of Th2-associated cytokines and late-phase inflammation, but did not produce IgE and IgG1 and did not undergo anaphylaxis [21]. We also recently showed that IL-4 KO mice were deficient in IgE and IgG1 production. The lack of IgE and IgG1 led to the protection of IL-4 deficient mice from food-induced anaphylaxis. We demonstrated that the critical source of IL-4 in this peanut allergy model was the naïve CD4 T cell, which effected autocrine/paracrine IL-4 signalling to amplify and stabilize the Th2 state [22].

Although the molecular mechanisms that mediate Th2 polarization have been extensively reviewed [10,23], the incipient events that predispose the immune system to launch a Th2 immunity program are less understood, particularly as it refers to innocuous proteins (i.e., food allergens). Recently, we discovered that CD4 T cells carry multiple potentials and that the acquisition of a Th2 identity largely depends on the environmental cues sensed by the DC as well as the subsequent interactions that take place during allergen presentation to the impressible naïve T cell [20].

## 3. On the Innocuousness of Food Allergens

The term antigen refers to the ability of certain molecules to induce antibody generation. When an IgE response is generated against an antigen, it qualifies as an *allergen*. Allergens that induce IgE production and IgE-mediated allergic reactions are defined as *complete* (e.g., Der p 1, Ara h 2) [24,25]; this contrasts with those not implicated in the sensitization process but are still recognized by IgE (e.g., cross-reactive allergens) [26]. The description of the hallmark features that constitute an allergen has been a recurrent area of research since the 1970s [27]. Some researchers have argued that, given the right conditions, any antigen can become an allergen [28]. However, allergen sequence analysis has demonstrated that a limited amount of protein families (<2%) contains most of the known allergens (>700) [29], and similar findings were also reported for food allergens of plant [30] and animal origin [31]. These data indicated the existence of common structural, biochemical, and functional characteristics of food allergens in Th2 responses.

Significant effort has been dedicated to understand the biochemical alterations of allergens brought about by food processing (e.g., heat treatment, Maillard reaction, food matrix effects, etc.) as well as their digestibility [32,33,34,35,36] under the premise that food allergens meet the immune system first in the gastrointestinal tract. This effort ultimately concluded that the allergenicity of a given protein could not be predicted on the basis of its stability and/or digestibility alone. These factors may contribute to overall allergenicity and can be useful in the design of superior allergen preparations for immunotherapy [37] (e.g., heated and ovomucoid-depleted egg white [38,39]). However, both the detection of food allergens in the bloodstream following oral ingestion [40,41,42,43], likely via absorption through the oral mucosa [43] and the discovery of the skin as a site for allergic sensitization in humans [44,45,46,47] imply that intact food allergens reach the immune surveillance system. Therefore, the tissue microenvironment where the allergen and the immune system meet may dictate whether a Th2 response is elicited.

The immunosurveillance system largely relies on receptors that recognize pathogen- or damage-associated molecular patterns (PAMPs or DAMPs/alarmins, respectively) [48]. The innate immunostimulatory properties associated with certain food allergens may involve signalling via PAMP or DAMP receptors [49,50,51]. For example, TLR4, a PAMP receptor that recognizes LPS, has been linked to several allergic diseases [52]. Food allergens with lipid-binding properties (e.g., 2S albumins, non-specific lipid-binding proteins, prolamin storage proteins, etc.) may engage TLR4 signalling by binding to LPS [53]. In other cases, allergens can directly bind to the TLR4/MD-2 complex, as has been reported for α-amylase/trypsin inhibitors [54], which are allergenic proteins of the prolamin family [55]. Through the activation of pattern recognition receptors (PRRs), allergens may create an inflammatory environment that facilitates sensitization. Specifically, invariant natural killer T cells recognize cow’s milk sphingolipids presented via CD1d on antigen-presenting cells (APCs) and promote an environment prone for Th2 responses [56]. Interestingly, data generated with models of intragastric sensitization to β-lactoglobulin and peanut demonstrated that TLR4 was not required for IgE-responses [57,58,59]; however, these studies notably used the adjuvant cholera toxin (CT), which may have compensated for the lack of TLR4 [60,61].

C-type lectin receptors are a family of PAMP receptors that bind carbohydrate ligands, which are a common constituent of food allergens. Within this family, the mannose receptor was shown in vitro to mediate Ara h 1 (a major peanut allergen) internalization by human DCs [62]. Additionally, DC-SIGN was critical for Ara h 1-mediated Th2-polarization, which was lost upon Ara h 1 deglycosylation [63]. Notably, culturing DCs with antigen-coupled Lewis-x trisaccharides suppressed IL-12 production (a pro-Th1 cytokine), which is likely a relevant mechanism of Th2-polarization induced by glycans [64]. In addition, the scavenger receptor A (SR-A) family, which is specific to modified low-density lipoproteins [65], has been shown to drive uptake and MHC II presentation of OVA to OT-II CD4 T cells [66]. SR-A also mediated DC uptake of glycated OVA, which induced higher CD4 T cell responses [67] and IgE production than native OVA [68]. Evidence suggests that inherent and/or induced glycosylation of food allergens potentiates their allergenicity [62,63,64,65,66,67,68]. For example, advanced glycated end products, which are frequently produced during food processing and cooking, share their receptors with high-mobility group box protein 1, an alarmin that promotes Th2 immunity [69,70]. This indicates that food allergy might be associated with high dietary advanced glycation end-products and pro-glycating dietary sugars that mimic alarmins [69,70]. However, no evidence has demonstrated that advanced glycation end products initiate food allergy; consequently, their biological relevance on the immunostimulatory properties of food allergens remains to be elucidated.

The activation of Th2 responses by allergens via DAMP receptors can involve enzymatic activity. For example, papain (papaya proteinase I) is a cysteine protease with similar enzymatic activity to that of Act d 1 from kiwi or Ana c 2 (bromelain) from pineapple. Papain-like proteases can disturb the epithelial barrier, cause cellular damage, and the release of alarmins [52]. For example, the exposure of mouse airway epithelial cells to papain and bromelain induced the alarmins uric acid (UA), thymic stromal lymphopoietin (TSLP), and IL-33 which facilitated the generation of Th2 immunity against OVA [71]. A recent study suggested that the protease activity of papain might drive the sensitization process, reporting a reduced IgE production after subcutaneous protease inhibitor-treated papain [72]. However, another study showed that mice epicutaneously sensitized to protease inhibitor-treated papain had IgE responses comparable to those induced with the active form [73]. Accordingly, although the role of protease activity in sensitization to aeroallergens is well characterized, its role in food allergy is not yet clear [74]. Further, there is no evidence that major food allergens, such as peanut, tree-nuts, fish, and shellfish, contain enzymatic activity.

Are food proteins innocuous? On the one hand, some foods can be sensed and internalized by innate immune cells and some, especially those with protease activity, can cause damage [73,75]. On the other hand, there is extensive evidence that exposure to foods in the vast majority of individuals induces tolerance. The effectiveness of this process is attested by the prevalence of self-reported food allergy (7%) in comparison to its much lower actual prevalence, as demonstrated by gold standard diagnostic tests, such as a controlled food allergen challenge [76,77,78,79,80]. Experimental models substantiate that ingestion, inhalation, or topical exposure to food antigens under homeostatic conditions is either ignored by the immune system or defaults to the induction of immunological tolerance [81,82,83,84,85,86,87]. Moreover, both the high prevalence of multiple food allergies within food-allergic patients and the evidence that over 170 foods can trigger allergic reactions in humans point towards an allergen-independent, mechanism-driving, allergic sensitization [75,88]. In summary, inherent allergenicity of foods likely represents a minor contribution to the development of food allergy. Accordingly, the ability of a given food to become an allergen may largely depend on concomitant events that subvert tissue homeostasis and produce signals that facilitate an immune response against a bystander food antigen.

## 4. External Subverters of the Steady State

Allergic sensitization is a clinically silent process and, therefore, exceedingly difficult to study in humans [89]. From this perspective, murine models have become powerful tools to identify the immunological mechanisms underlying allergic sensitization. On the basis of these experimental studies, it is possible to generate an immune response to any food when that food is administered along with an “adjuvant”, which is a term derived from the Latin “*adjuvare*”, meaning ‘to aid’ [90]. Certain adjuvants alter tissue homeostasis to establish conditions that result in non-specific, innate Th2 priming to bystander allergens.

The feeding or intragastric gavage of a food by itself results predominantly in immune tolerance in mice [91]. This homeostatic response can be subverted when such food is administered alongside adjuvants such as CT [21,92], which induces a strong immune response towards itself that extends to the bystander allergens. It is in the context of the CT-induced response that food allergens are taken and processed by immunosurveillance cells. Accordingly, CT has been extensively employed in models of oral sensitization to food allergens including peanut [21,93], egg [39,94,95], and milk [58,96], among others [97]. Some of the immune-stimulating effects of CT have been characterized. For example, CT has been shown to induce maturation and activation of DCs and promote their subsequent migration to the draining lymph nodes [98]. In a similar fashion, Shreedhar et al. reported that CT induces migration of the DCs from the subepithelial dome region of the intestine to the T- and B- cell zones of the Peyers’ patches [99]. Specifically, Gustafsson et al. demonstrated that the ability of CT to activate DCs is rooted in its interaction via GM-1 ganglioside and is independent of direct activation of intestinal epithelial cells [61]. In addition, we recently demonstrated that CT induces intestinal eosinophil degranulation and release of the alarmin eosinophil peroxidase (EPO), which is critical for DC priming and allergic Th2 sensitization in the gut [100]. We have also shown that mice depleted of UA or deficient in IL-33 were protected from anaphylaxis using an intragastric sensitization protocol with CT [59,101]. Staphylococcus aureus, a major food contaminant which produces enterotoxin B (SEB), is another adjuvant used in models of oral sensitization [102,103]. SEB has been shown to cause Th2 polarization by upregulating the co-stimulatory molecule TIM-4 on intestinal DCs [104]. In addition, SEB upregulates IL-33 [105,106,107]. Additionally, MyD88-/- mice, which cannot signal through the IL-33 pathway (among others), were resistant to the effects of SEB.

Although the intragastric route has been frequently used to induce food allergy in mice, as previously mentioned, increasing evidence suggests that the skin may be a relevant route of sensitization to food allergens in humans. This has prompted the use of experimental models of epicutaneous sensitization to foods. Allergic sensitization is usually achieved by causing a barrier disruption, typically through tape stripping (TS), prior to the placement of the allergen on the skin (sometimes through a patch) [108,109,110]. TS, which could be considered an external subverter, induces mechanical injury, damage, and local release of IL-33 [111], UA [59] and TSLP [108,112,113], etc. These alarmins are required for allergic sensitization through the priming of DCs to elicit a Th2-polarized immune response [114,115]. Notably, mice deficient in UA were protected from allergic sensitization and thus clinical reactivity [59]. The concept that alarmins are produced downstream to the external subverters (e.g., TS) and are sufficient to induce sensitization has been explored. For example, we induced allergic sensitization in mice epicutaneously exposed to peanut (no TS) in addition to subcutaneous administration of monosodium urate (i.e., UA) crystals [59]. Similarly, persistent induction of TSLP (by transgenic overexpression, injection of recombinant protein, or repeatedly stimulated epithelium) at a barrier site caused inflammation and promoted sensitization to bystander antigens [116,117,118].

The term ‘adjuvant-free’ has been used in reference to certain models of sensitization [110,117,119] in which the immune response is claimed to be exclusively triggered by the food allergen. Although these models appear to be ‘adjuvant-free’, the molecular signature of the sensitization phase is characterized by the presence of damage and/or DAMPs. Dolence et al. demonstrated that airway exposure to peanut flour, in the absence of any adjuvant, induced peanut-specific IgE and anaphylaxis upon challenge [120]. However, this exposure induced the alarmins IL-1α and IL-1β, indicating that the ectopic exposure of peanut protein resulted in airway damage. Likewise, Tordesillas et al. reported an ‘adjuvant-free’ epicutaneous model which required the use of hair-removing depilatory cream. However, this procedure was also associated with the release of alarmins and was reliant on the IL-33-ST2 interaction in the skin-draining lymph nodes, thus intimating that the depilatory cream had inherent adjuvant effects. Interestingly, the same model was used for epicutaneous immunotherapy, suggesting that this exposure could be both sensitizing and tolerizing in different contexts [119].

## 5. Converging Pathways Leading to Th2 Sensitization

**Figure 1:**
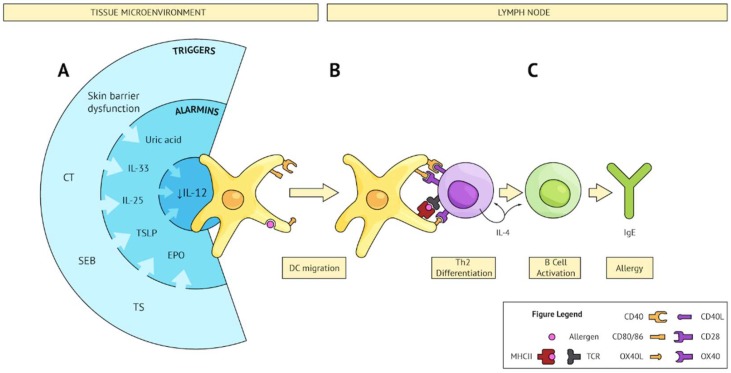
Converging pathways leading to Th2 sensitization. (**A**) Several triggers (external; e.g., CT, SEB, TS, or internal; e.g.*,* skin barrier dysfunction) share the ability to subvert the microenvironment and produce an alarmin signature which converges on IL-12 inhibition on DCs and upregulation of Th2 polarizing co-stimulatory molecules. These activated DCs sample bystander allergens and migrate to secondary lymphoid tissue. (**B**) Activated DCs present allergen to cognate naïve CD4 T cells and provide co-stimulation resulting in Th2 differentiation. (**C**) Th2 cells activate allergen-specific B cells, leading to IgE class switching, plasma cell differentiation, and production of allergen-specific IgE.

A diverse array of external subverters can specifically prime the innate immune system such that concomitant exposure to a food allergen results in a common outcome: an allergen-specific Th2 immune response. The path from diversity to commonality is illustrated in Figure 1. It proposes that certain subverters (e.g., CT, SEB, TS, etc.) establish a first degree of convergence, characterized by tissue damage and the production of an alarmin signature that may include IL-33, TSLP, IL-25, UA crystals, and EPO [59,101,117,121]. Although these alarmins are individually able to activate DCs, the question remains as to why the ultimate outcome would be a Th2 response. In such contexts, a second degree of convergence applies. Evidence suggests that many of these tissue-derived alarmins can inhibit IL-12 production. Thus, the attendant inhibition of IL-12 facilitates the adaptive immune response to become Th2-polarized [122]. In this regard, TSLP has been shown to promote Th2 immunity by conditioning DCs to downregulate IL-12 production and upregulate the costimulatory molecule OX40L [123]. IL-25 and IL-33 have been shown to induce DC OX40L [101,110]. Importantly, IL-33 has been reported as critical for Th2 polarization as well as the induction of allergen-specific IgE and anaphylaxis in models of both gut and skin allergic sensitization [101,110]. In addition to OX40L [124], the costimulatory molecules CD40 [125,126] and CD80/86 [127,128] on the DC are required for Th2 differentiation. As previously mentioned, UA and EPO have been reported as critical in allergic sensitization because their removal prevented it, even in the presence of an adjuvant like CT. Although experimental models of Th2 sensitization used adjuvants to cause a cascade of signalling that resulted in IL-12 inhibition, it appears that this mirrored the natural Th2 immune response to parasites. Notably, *Leishmania major* has been shown to block IL-12 production in macrophages and DCs [129]. The requirement of alarmins in addition to IL-12 inhibition and co-stimulation enhances the *unified model* of Th2 sensitization proposed by Liu et al. [130].

The modulation of the innate and eventually adaptive immune response through external Th2 subverters, such as adjuvants or parasite-derived products, explains fundamental principles of Th2 priming. However, which subverters (adjuvants) facilitate the development of food allergy in humans remains obscure, largely a result of the silent nature of sensitization. A bird’s eye view of the process might be enlightening. Although it is highly unlikely that CT, a product of *Vibrio cholera*, plays a significant role in the development of human food allergy, CT is a member of the AB5 family of bacterial toxins [131]. Toxins belonging to this family are produced by other bacteria, including *Escherichia coli*, *Campylobacter jejuni*, *Bordetella pertussis*, and *Shigella dysenteriae*, among others [131]. In addition, SEB is a product of *Staphylococcus aureus*; mycotoxins produced by *Fusarium* species, the most frequent contaminants of crops, have been shown to promote sensitization to food allergens [132]. Therefore, nature affords sufficient opportunities for the exposure to toxins with the capacities documented for CT. Further, both Th2 cytokines, such as IL-4, and allergens, such as pollen, also have the capacity to inhibit IL-12 as well as directly stimulate Th2 polarization in DCs [133,134]. Lastly, several external subverters capable of indirectly perturbing the internal milieu may establish conditions conducive to allergic sensitization. For example, bacterial exposure early in life, antibiotic and antacid abuse during infancy, or birth through caesarean section have been associated with increased prevalence of food allergy, presumably a result of alterations to the homeostatic microbiota [135,136,137,138,139].

## 6. Internal Subverters of the Steady State

The genetics of allergic diseases have been long studied. In part, this is a result of the hereditability of numerous allergic diseases (i.e., allergic rhinitis, asthma, atopic dermatitis (AD)) [140]. The development of AD in childhood is known to be a major risk factor for later developing other allergic diseases such as asthma and, notably, food allergy (known as the atopic march) [141,142,143]. For example, a recent systematic review determined an odds ratio of 6.2 for self-reported food sensitization in AD versus non-AD children [141]. One gene associated with AD and food allergy is filaggrin, which maintains skin barrier integrity [144,145]. A meta-analysis by van den Oord et al. found that filaggrin gene defects were highly associated with AD development and allergic sensitization [146]. In addition, neonatal mice with filaggrin mutations (Ft+/- Flgft Tmem79ma) developed allergic sensitization to a low dose of house dust mite plus peanut whereas wild-type animals did not [147]. AD has also been shown to cause tight junction dysfunction and, accordingly, an aberrant stratum corneum, which would likely facilitate epicutaneous Th2 sensitization [148]. Furthermore, a SPINK5 gene variant affecting epidermal integrity has also been associated with allergic predisposition in infants [45].

Additional mechanisms can lower the immune activation threshold and increase the risk of food allergy, as it has been shown for polymorphisms in IL-10 [149], IL-13 [150,151], IL-4 [150], IL-4Rα [150], as well as STAT6 [152]. Likewise, polymorphisms in CD14 as well as a variant of IPEX caused by deletions in the non-coding region of FOXP3 (the master transcription factor of Treg cells) [153] are both associated with food allergy. Several of these polymorphisms have been investigated in experimental models. For example, IL-4Rα transgenic mice [154,155] exhibited a lower threshold of activation for a Th2 immune response compared to wild type animals, thus enabling IgE responses without external subversion. Lastly, primary immune deficiencies (i.e., selective IgA deficiency, hypogammaglobulinemia) as well as hyper-responsive innate immune responses have been associated with the development of allergic disease [139,156,157,158,159,160]. Although a comprehensive analysis of the genetics of food allergy has been reviewed elsewhere [161], these examples provide insight into the contribution of several genetic components for the development of Th2 immunity against foods. It is manifest that genetics play a role in the development of food allergy through alterations in homeostasis, which increases the risk of mounting IgE responses against food allergens. However, only a small fraction of patients with these mutations develop allergic diseases, indicating that their role in initiating food allergy is limited.

## 7. Concluding Remarks

We have proposed that neither inherent food allergenicity nor individual genetics play major roles in the induction of allergic sensitization. Thus, it stands to reason that the conceptual understanding for the initiation Th2 immunity towards food allergens merits an alternative paradigm.

**Figure 2:**
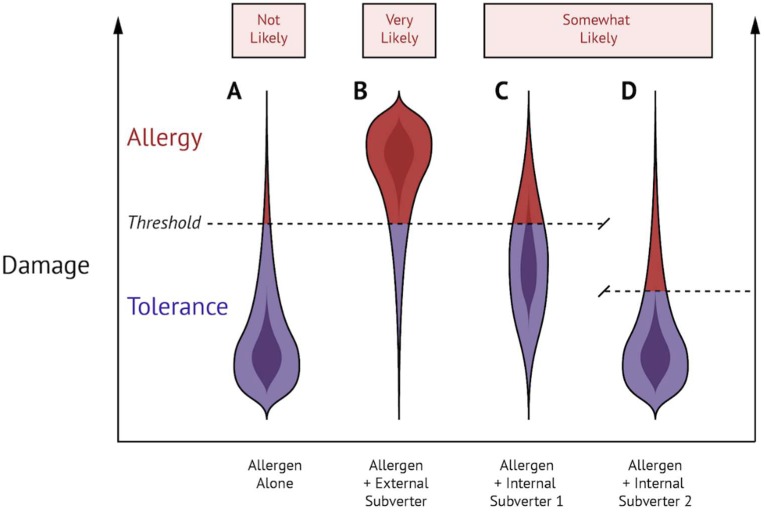
External and internal subversion of the steady state can lead to food allergic sensitization. (**A**) Allergen exposure alone results in damage that is ‘not likely’ sufficient to cause food allergy. (**B**) Exposure to allergen in combination with a Th2-inducing external subverter results in damage that is ‘most likely’ sufficient to cause allergy. (**C**,**D**) Exposure to allergen in combination with an internal subverter that either 1 (increases basal level of damage) or 2 (decreases the threshold of Th2 activation) is ‘somewhat likely’ sufficient to cause allergy.

Although Claude Bernard first described the concept of homeostasis in 1865, the term was coined by Walter B. Cannon in 1962. It refers to the ability of an entity, whether a cell, an organ, or an organism, to maintain an internal equilibrium despite outside changing conditions. This equilibrium, achieved by a system of feedback controls, is essential for the proper functioning of the entity. From this perspective, a disease is a loss of such equilibrium. In the context of Th2 immune responses against food allergens, disequilibrium can be achieved by external or internal perturbations that either activate or lower the threshold of activation of the system. Although, in immunology, the term adjuvant typically refers to any substance that activates the immune system, we suggest that any condition or intervention which tempers with the steady state could be considered an adjuvant. This phenomenon is illustrated in Figure 2 in which we propose the potential likelihood of allergic sensitization based on the varying types of subverters present in combination with the allergen.

Regardless of priming conditions and the nature of the food allergen, a convergence toward key checkpoints delineates a pathway leading to Th2 sensitization. A caveat to this paradigm is that the trajectory of food allergic disease is heterogeneous in terms of persistence of the disease, clinical reactivity, and response to treatment. At this point, the extent to which initial sensitizing conditions influence the trajectory of food allergic disease remains enigmatic.

Although food allergy has increasingly become a major health and economic concern, it remains a relatively infrequent outcome given our frequent exposure to foods. We suggest that food allergy arises as a result of the coincidence in time and space with several seemingly unconnected incidents that destroy the state of equilibrium. Exposure to a certain food in the context of a concomitant event that induces tissue damage and the subsequent production of an alarmin signature capable of inhibiting IL-12 production not only overcomes the steady state but also directs the immune system towards a Th2 pathway. The likelihood of this outcome is presumably enhanced by other contributing incidents, such as the enzymatic activity of some allergens and the lowering of internal thresholds due to genetic mutations. Beyond biology, social practices such as the abuse of antibiotics in infancy, misguided recommendations regarding the delayed introduction of foods (notably peanut) into the diet of infants, manufacturing processes that enhance food allergenicity, or the consumption of foods from contaminated crops likely play a contributory impetus. Arguably, the development of food allergies is the result of an infrequent “perfect storm”.
